# Antenatal identification of early- and late-onset fetal growth restriction and the possible impact of the introduction of cerebroplacental ratio: Effect on perinatal and childhood outcome

**DOI:** 10.1371/journal.pone.0325906

**Published:** 2025-06-18

**Authors:** Emma Hertting, Lotta Herling, Pelle G. Lindqvist, Eva Wiberg-Itzel

**Affiliations:** 1 Department of Clinical Science and Education, Karolinska Institutet, Södersjukhuset, Stockholm, Sweden; 2 Department of Obstetrics and Gynecology, Södersjukhuset, Stockholm, Sweden; 3 Center for Fetal Medicine, Pregnancy Care and Delivery, Karolinska University Hospital, Stockholm, Sweden; 4 Department of Clinical Science, Intervention and Technology, Karolinska Institutet, Stockholm, Sweden; Imperial College London, UNITED KINGDOM OF GREAT BRITAIN AND NORTHERN IRELAND

## Abstract

**Objective:**

To investigate the effect of antenatal identification of small for gestational age (SGA) fetuses on perinatal and childhood outcomes, separately analyzing early- and late-onset fetal growth restriction (FGR).

**Materials and methods:**

A register-based cohort study of all newborns born SGA, delivered in Stockholm in 2014 and 2017, n = 5499. Ultrasound reports of fetuses born SGA were reviewed and fetuses identified as SGA with ultrasound before birth were further defined as early- or late-onset FGR according to established criteria. Data from the medical chart for maternity and delivery was linked to nationwide Swedish registers. Adverse outcomes for antenatally non-identified SGA/FGR newborns and fetuses identified as early- or late-onset FGR were compared using logistic regression models. A composite outcome, severe adverse outcome, was constructed and defined as at least one of the following: stillbirth, severe newborn distress, severe neonatal outcome, severe childhood outcome. Individual components of the composite outcome were analyzed as secondary outcomes.

**Results:**

Identified early-onset FGR fetuses had an increased risk for severe adverse outcome, compared to non-identified SGA/FGR (aOR 1.81, 95% CI 1.25–2.61), in contrast to late-onset FGR fetuses (aOR 1.14, 95% CI 0.78–1.67). Identified early-onset FGR had a decreased risk of stillbirth (aOR 0.47, 95% CI 0.23–0.96), an increased risk of severe newborn distress (aOR 2.80, 95% CI 1.79–4.39) and severe childhood outcome (aOR 3.00, 95% CI 1.51–5.94), compared to non-identified SGA/FGR. Identified late-onset FGR was only associated with an increased risk of severe childhood outcome (aOR 1.91, 95% CI 1.04–3.52).

**Conclusion:**

Identified early-onset FGR fetuses benefited from identification with a decreased risk of stillbirth at the price of an increased risk for severe newborn and childhood outcomes. For late-onset FGR the advantages were undetectable; identification was associated with an increased risk for severe childhood outcome, while the negative association with stillbirth did not reach significance.

## Introduction

Fetal growth restriction (FGR) is associated with increased risk for stillbirth, perinatal morbidity and mortality, cerebral palsy, neurodevelopmental disorders and cardiovascular disease later in life [1–5].

Screening for small for gestational age (SGA) aims to identify fetuses at risk for FGR, in order to decrease mortality and morbidity. The benefits of identifying SGA have been described in previous studies [6,7]. We recently showed that the effect of identification of SGA differed between outcomes; non-identified SGA fetuses had a 5-fold increased risk for stillbirth, a slightly increased risk for severe newborn distress, but a decreased risk for severe childhood outcomes, compared to identified SGA fetuses [8]. No previous studies have investigated if the effect of identification on perinatal and childhood outcomes differs between early-onset FGR (E-FGR) and late-onset FGR (L-FGR). Compared to L-FGR, E-FGR is strongly associated with preeclampsia, often linked with a more pronounced weight deviation, and the incidence of severe perinatal morbidity and mortality is higher. There is research guiding us on how to monitor and when to deliver E-FGR [9]. For L-FGR, we still lack enough evidence to decide the optimal time point for delivery. The DIGITAT trial showed no important differences in perinatal and 2-year outcomes, comparing immediate induction vs expectant management in late SGA pregnancies (> 36 weeks of gestation) [10,11]. However, other studies have shown that children born early term have poorer outcome than children born late term [12–14]. Hence, there is reason to believe that the effect of being identified as SGA/FGR before birth may differ between E-FGR and L-FGR. The aim of this study was to estimate the effect of being identified as SGA/FGR before birth on perinatal and childhood outcomes, analyzing E-FGR and L-FGR separately. Furthermore, we evaluated the introduction of a mandatory assessment of the cerebroplacental ratio (CPR) in late SGA pregnancies.

## Materials and methods

This was a secondary analysis of a Stockholm based cohort, comprising all singletons, without known chromosomal aberrations or structural abnormalities and with a gestational age at delivery between 22 + 0 and 43 + 0 weeks, born in 2014 and 2017. The study cohort, the setting and the employed registers have been described elsewhere [8]. Briefly, cases were identified by the regional computerized medical chart for maternity and delivery (Obstetrix, Cerner Sverige AB, Lund, Sweden), which contains data on maternal and infant anthropometrics, medical diagnoses, and the personal identity number (PIN), uniquely assigned to all citizens. The PIN was used to cross-link the data to the Swedish National Patient Register, The Swedish Cause of Death Register, The Swedish National Quality register and Statistic Sweden’s Longitudinal Integrated Database for Health Insurance and Labor Market Studies (LISA), for information on later disease and/or death of the offspring and maternal socioeconomic data [15–19]. For the purpose of the present study, only infants born SGA were included. SGA was defined as ≤ −15% of the mean weight, which approximately corresponds to <10^th^ percentile.

In Sweden, 92% of all pregnancies are dated by ultrasound, 6.2% based on the date of embryo transfer, 1.2% by the last menstrual period and 0.2% by other methods [20]. Ultrasound dating was performed in first or early second trimester, based on a measurement of the crown rump length between 45−85 mm or a biparietal diameter between 21 and 55 mm [21]. A selective screening was used to identify fetuses at risk for FGR, i.e., ultrasound in the second and/or third trimester was performed only in high-risk pregnancies, if pregnancy complications occurred or if there was a deviation in the symphysis fundal height. Ultrasound and Doppler examinations were performed by specialists, trained and examined by the Swedish Society of Obstetrics and Gynaecology. Estimated fetal weight (EFW) and weight-deviation from the expected mean weight were calculated, based on established formulas [22,23]. An EFW of ≤ −15% of the mean, approximately corresponding to < 10^th^ percentile, required further investigations. All units used the same internet-based guidelines for the screening and management of SGA and FGR, see S1 Appendix. In 2015, a compulsory Doppler examination of the middle cerebral artery in fetuses ≥ 34 + 0 weeks with an EFW of ≤ −15% was added to the guidelines. The aim was to improve the diagnostics of SGA vs L-FGR according to new international consensus criteria, which included CPR (pulsatility index in the middle cerebral artery/ pulsatility index in the umbilical artery) [24]. A recommendation to consider delivery for fetuses with a CPR < 5^th^ percentile and a gestational age of > 38 + 0 weeks was added. The study cohort covers one year before and one year after this change, which enabled an evaluation of the introduction of CPR in late SGA pregnancies.

### Exposure

Exposure was defined as identified as E-FGR or L-FGR before birth. The comparison group was SGA/FGR newborns who were not identified with ultrasound before birth. For L-FGR, the comparison group was restricted to include non-identified SGA/FGR delivered after 32 + 0 weeks of gestation. It has previously been shown that a gestational age cut-off of 32 weeks at diagnosis maximizes the difference between E-FGR and L-FGR [25]. All ultrasound examinations of fetuses born SGA were reviewed. Fetuses were considered identified if at least one ultrasound examination presented an EFW of ≤ −15% (≈ < 10^th^ percentile) and non-identified if no ultrasound was performed after the routine anomaly scan, or if the performed ultrasound resulted in an EFW of> −15%. Fetuses with an EFW of ≤ −15% were classified as E-FGR or L-FGR, according to internationally established consensus criteria, see S1 Table, where early corresponds to detection before 32 + 0 and late ≥ 32 + 0 weeks of gestation [24]. Fetuses with an EFW of ≤ −15%, but ≥ −22% (approximately corresponding to < 10^th^, but ≥ 3^rd^ percentile) with complete and normal Doppler examinations were defined as identified SGA. If the Doppler examinations were incomplete, i.e., at least one Doppler examination needed for the classification was not performed, cases were defined as “unclassified” (UC). Identified SGA and UC fetuses were included in the analyses and reported as separate groups.

### Outcome

The outcome was *severe adverse outcome*, a composite outcome, defined as at least one of the following: 1) stillbirth, 2) severe newborn distress, 3) severe neonatal outcome or 4) severe childhood outcome. Each subgroup of outcomes was then analyzed separately. *Stillbirth* was defined as intrauterine death at ≥ 22 + 0 weeks of gestation or death during delivery. *Severe newborn distress* was defined as at least one of the following: An Apgar score of < 4 at 5 minutes, a pH of < 7.0 in the umbilical artery or resuscitation activities of > 10 minutes. *Severe neonatal outcome* was defined as at least one of the following: hypoxic ischemic encephalopathy grade 2–3, necrotizing enterocolitis, neonatal seizures, intraventricular hemorrhage grade 3–4, bronchopulmonary dysplasia or death (< 1 year old). *Severe childhood outcome* was defined as at least one of the following: cognitive impairment, motor impairment, cerebral palsy, hearing impairment, visual impairment or death (1–3 years old). The outcome data were chosen in accordance with a Delphi procedure consensus document on a core outcome set for FGR [26].

Outcome data were extracted from 1) the computerized medical record for maternity and delivery (Apgar score, umbilical artery pH and stillbirth), 2) the Swedish Neonatal Quality Register (resuscitation activities of > 10 minutes, hypoxic ischemic encephalopathy grade 2–3, necrotizing enterocolitis, neonatal seizures, intraventricular hemorrhage grade 3–4, bronchopulmonary dysplasia), 3) the Swedish National Patient Register (motor impairment, cognitive impairment, cerebral palsy, hearing impairment and visual impairment), and 4) the Swedish Cause of Death Register (infant and childhood death). The National Patient Register was requested to provide data on the following ICD-10 codes of the study population from 2014 to 2020: G80-G82, F82, F70-F73, F78-F80, F83, F84, H54 and H90.

### Covariates

To minimize confounding bias, a directed acyclic graph was constructed, exploring factors influencing the association between antenatal identification of FGR and severe adverse outcome (S1 Fig). The final minimal sufficient set for confounder adjustment included maternal age, body mass index (BMI), smoking status, socioeconomic status, parity, diabetes, preeclampsia/hypertension, other diagnoses associated with preterm birth and weight deviation at birth. Educational level was used as a proxy for socioeconomic status and defined as low (< 9 years), medium (9–12 years) or high (> 12 years). BMI was based on measured weight and self-reported height in the first trimester. Smoking was defined as smoking in early pregnancy yes/no and hypertension as a blood pressure of >140/90 at two separate measurements before or during pregnancy. Preeclampsia was defined as pregnancy-related hypertension and proteinuria ≥ 300 mg/24h after 20 weeks of gestation. Diabetes included diabetes mellitus type 1, type 2 and gestational diabetes, defined as a fasting venous plasma glucose of ≥7 mmol/l and/or a 2-hour value of ≥9 mmol/l after a 75g oral glucose tolerance test. Other diagnoses associated with preterm birth were defined as at least one of the following: spontaneous preterm delivery, preterm premature rupture of membranes, placenta accreta spectrum, placenta previa, ablatio placentae. Weight deviation at birth was calculated based on the actual birthweight as (birthweight – expected birthweight by gestational age)/expected birthweight by gestational age, and expressed as a percentage. Expected birthweight by gestational age was calculated using the formula for intrauterine expected fetal weight [23].

Information on maternal age, BMI, smoking status, parity, preeclampsia, hypertension, diabetes, other diagnoses associated with preterm birth (spontaneous preterm delivery, preterm premature rupture of membranes, placenta previa, ablatio placentae and, placenta accreta spectrum) and birthweight were obtained from the medical chart for maternity and delivery and educational level from the LISA register.

### Statistical analyses

Maternal characteristics, pregnancy complications, start and mode of delivery, pregnancy outcomes and severe perinatal and childhood outcomes were compared between antenatally non-identified SGA/FGR newborns and newborns identified as E-FGR, L-FGR, SGA or UC before birth. Continuous variables were presented as medians with interquartile range (IQR) and categorical variables as numbers and proportions. The comparisons between the continuous variables were made with the Mann-Whitney U test and with chi-square test for the categorical variables. Fisher’s exact test was used for categorical variables if the expected count was less than five.

Logistic regression analyses were used to assess the association between being identified as E-FGR or L-FGR and severe adverse outcome. The exposure variable was categorized as SGA/FGR non-identified before birth (reference) and fetuses identified as 1) E-FGR, 2) L-FGR, 3) SGA and 4) UC before birth. The outcome (dependent) variable was categorized as severe adverse outcome, yes or no. Covariates were the previously identified possible confounders, i.e., maternal age, BMI, smoking, educational level, parity, preeclampsia/hypertension, other diagnoses associated with preterm birth and weight deviation at birth. Diabetes was not included because of a negligible prevalence. Confounding variables were categorized as follows, based on clinical relevance: age > 35 years (yes or no), BMI (< 18.5, 18.5–24.9, 25–30, > 30), educational level (< 9 years, 9–12 years or > 12 years), smoking (yes or no), nulliparity (yes or no), preeclampsia/hypertension (yes or no), other diagnoses associated with preterm birth (yes or no) and weight deviation at birth (−15.0% to −22.0%, −22.1% to −28.0%, −28.1% to −33.0% or <−33.0%). Continuous variables were categorized due to a non-linear relation to the outcome. Crude odds ratios (cOR) for severe adverse outcome in relation to exposure were estimated. Then, adjusted odds ratios (aOR) were calculated, adding one confounding variable at a time.

To investigate the possible effect of being identified as E-FGR or L-FGR on stillbirth, severe newborn distress, severe neonatal outcome and severe childhood outcome separately, we performed logistic regression analyses with identical covariates and approach as described above.

All analyses were re-run with all possible two-way interactions.

Individuals with missing data on BMI, smoking and education were reported as separate categories of the variables and analyzed as such in the regression models.

Sensitivity analyses were performed. To evaluate the handling of missing data, we re-run the regression models 1) with imputed best-case, then worst-case scenario values for missing values on BMI, smoking and education and 2) as complete case analyses. Furthermore, we re-run the regression analyses 1) without categorizing the continuous variables and 2) only including covariates significantly affecting the associations.

To evaluate the introduction of a mandatory evaluation of CPR in late SGA pregnancies, an interaction analysis was performed, investigating the possible modifying effect of year of birth on the associations. For this purpose, the population was restricted to include only newborns with gestational age at birth > 32 + 0 weeks, in accordance with the gestational age cut-off at diagnosis for L-FGR [24,25]. In the logistic regression analyses we used identified SGA (no matter what diagnosis) as exposure and non-identified SGA/FGR as the reference.

Statistical differences were considered significant given a p-value of <0.05.

The data were analyzed using SPSS software (IBM SPSS Statistics for Windows, Version 26.0, Armonk, NY: IBM Corp).

### Ethical approval

This study was approved by the Swedish Ethical Review Authority (2019–04920, 2020-02-12, 2020–05295, 2020-11-06 and 2021–00315, 2021-02-11). The data were analyzed anonymously, oral or written consent was not required. The authors had no access to information that could identify individual participants. The data were accessed for research purposes the 24^th^ of August 2022.

## Results

**Fig 1:**
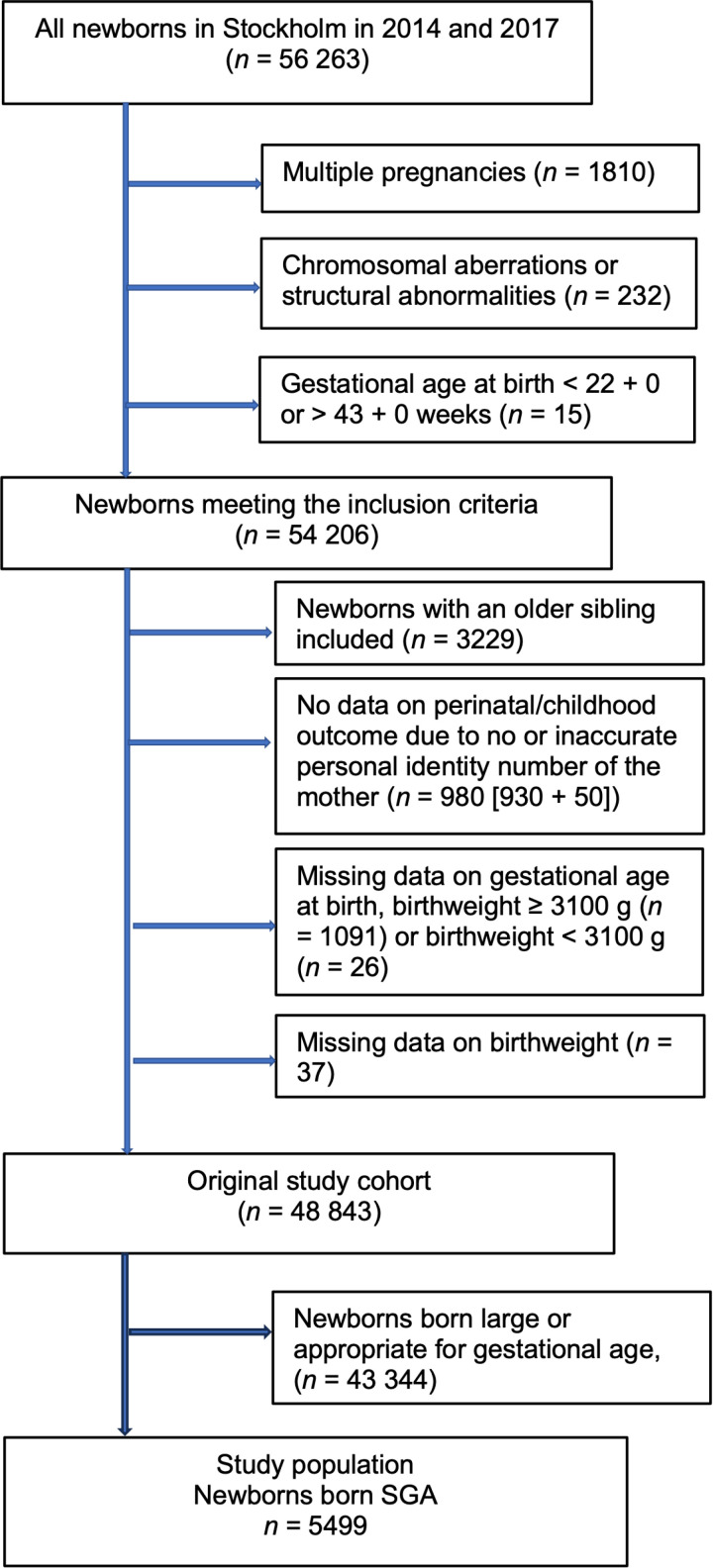
Flowchart of the study population.

**Fig 2:**
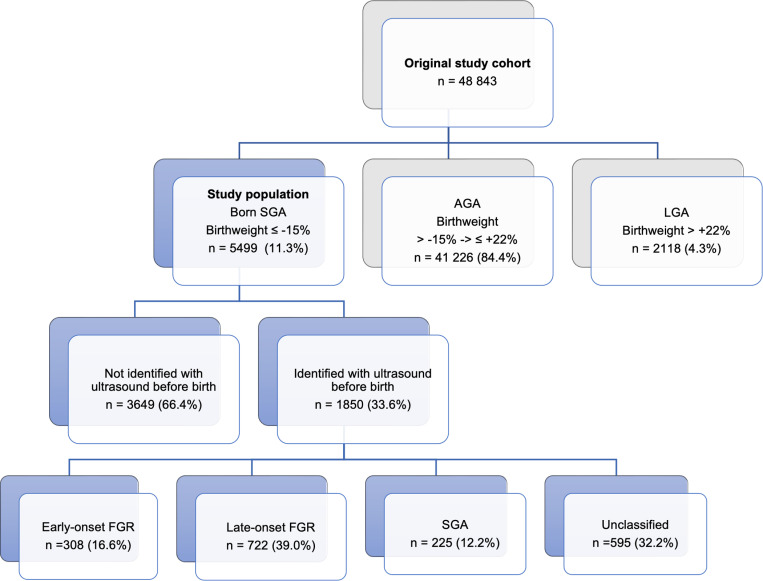
Details on study population. Number and proportions of newborns born appropriate for gestational age (AGA), large for gestational age (LGA) and small for gestational age (SGA). Numbers and proportions of SGA newborns identified and not identified by ultrasound as ≤ −15% before birth. Number and proportions of early- and late-onset FGR and SGA, according to the Delphi-criteria. Unclassified = unable to classify, due to incomplete Doppler examinations.

After the exclusion of 5363 subjects, the original study cohort consisted of 48 843 newborns (Fig 1). For the present study, the study population was restricted to include fetuses born SGA, n = 5499. The antenatal detection rate of SGA was 33.6%. Numbers and proportions of identified E-FGR, L-FGR, SGA and UC are presented in Fig 2.

Background characteristics and outcomes of non-identified SGA/FGR pregnancies compared to identified E-FGR and L-FGR are shown in Table 1. Maternal characteristics, pregnancy complications, start- and mode of delivery and pregnancy outcomes differed significantly between pregnancies diagnosed with E-FGR and non-identified SGA/FGR pregnancies in virtually all variables. L-FGR pregnancies presented, compared to non-identified SGA/FGR pregnancies, a higher proportion of smoking mothers (7.9% vs 4.5%), a higher proportion of hypertension (7.8% vs 3.6%), preeclampsia (11.8% vs 5.2%) and had higher proportions of mothers with low and high BMI. L-FGR pregnancies were more often induced to labor (49.7% vs 21.5%) and were to a higher extent delivered by emergency (24.1% vs 12.9%) and elective (10.7% vs 4.2%) cesarean section, compared to non-identified SGA/FGR pregnancies. The proportion of severe adverse outcome in non-identified SGA/FGR pregnancies was 6.0%, in identified E-FGR 23.7% and L-FGR 6.8%. For background characteristics and outcomes of fetuses classified as identified SGA and identified UC, compared to antenatally non-identified SGA/FGR, see S2 Table.

**Table 1:** Background characteristics and major outcome groups for non-identified SGA/FGR and identified early- and late-onset FGR.

	Non-ID SGA/FGR (reference), n = 3649	ID Early-onset FGR, n = 308	p-value	ID Late-onset FGR, n = 722	p-value
Maternal characteristics					
Age (years)	32.0 (28.0, 35.0)	32.0 (28.0, 37.0)	0.015	31.0 (28.0, 35.0)	0.222
>35	1062 (29.1)	117 (38.0)	0.001	199 (27.6)	0.404
BMI (kg/m^2^)	22.8 (20.8, 25.7)	23.7 (21.1, 27.6)	0.007	22.5 (20.4, 25.8)	0.069
<18.5	127 (3.5)	19 (6.2)	<.001	45 (6.2)	0.001
18.5-24.9	2268 (62.2)	142 (46.1)		422 (58.4)	
25-30	751 (20.6)	74 (24.0)		135 (18.7)	
>30	279 (7.6)	41 (13.3)		62 (8.6)	
missing	224 (6.1)	32 (10.4)		58 (8.0)	
Smoking (yes)	163 (4.5)	16 (5.2)	0.001	57 (7.9)	<.001
missing	191 (5.2)	31 (10.1)		50 (6.9)	
Nullipara (yes)	2249 (61.6)	145 (47.1)	<.001	450 (62.3)	0.726
Diabetes (yes)	16 (0.4)	7 (2.3)	0.001	10 (1.4)	0.006
Education					
≤ 9 years	385 (10.6)	35 (11.4)	0.251	81 (11.2)	0.476
10 to 12 years	1028 (28.2)	10 (32.5)		221 (30.6)	
>12 years	2108 (57.8)	160 (51.9)		395 (54.7)	
missing	128 (3.5)	13 (4.2)		25 (3.5)	
Start of delivery					
Spontaneous	2600 (71.3)	86 (27.9)	<.001	218 (30.2)	<.001
Induction	784 (21.5)	88 (28.6)		359 (49.7)	
Mode of delivery					
Spontaneous vaginal	2698 (73.9)	127 (41.2)	<.001	412 (57.1)	<.001
Instrumental vaginal	325 (8.9)	13 (4.2)		59 (8.2)	
Elective cesarean	154 (4.2)	42 (13.6)		77 (10.7)	
Emergency cesarean	472 (12.9)	126 (40.9)		174 (24.1)	
Pregnancy complication					
Hypertension	133 (3.6)	34 (11.0)	<.001	56 (7.8)	<.001
PE/HELLP	190 (5.2)	89 (28.9)	<.001	85 (11.8)	<.001
PTB-associated diagnoses^***^	171 (4.7)	35 (11.4)	<.001	46 (6.4)	0.057
Pregnancy outcome					
GA at delivery (days)	281 (274, 287)	258 (226, 274)	<.001	274 (264, 284)	<.001
Birth weight (gram)	2900 (2702, 3065)	2090 (1453, 2583)	<.001	2610 (2317, 2820)	<.001
Birth weight deviation (%)	−18.4 (−21.6, −16.5)	−26.7 (−32.7, 20.5)	<.001	−23.9 (−29.2, −19.9)	<.001
Severe adverse outcome^*^					
Severe adverse outcome^**^	220 (6.0)	73 (23.7)	<.001	49 (6.8)	0.439
Stillbirth	60 (1.6)	16 (5.2)	<.001	3 (0.4)	0.011
Severe newborn distress	105 (2.9)	46 (14.9)	<.001	27 (3.7)	0.216
Severe neonatal outcome	30 (0.8)	12 (3.9)	<.001	3 (0.4)	0.249
Severe childhood outcome	46 (1.3)	15 (4.9)	<.001	18 (2.5)	0.012

Continuous variables are presented as medians with interquartile range, categorical variables as numbers and proportions. SGA = small for gestational age, FGR = fetal growth restriction, Non-ID SGA/FGR = non-identified as ≤ −15% before birth, ID = identified as, BMI = body mass index, PE = preeclampsia, HELLP = Hemolysis Elevated Liver enzyme Low Platelet syndrome, GA = gestational age, PTB = preterm birth.

* Major outcome groups

** At least one of the below.

*** At least one of the following; spontaneous preterm birth, preterm premature rupture of membranes, placenta previa, placenta accrete spectrum, ablatio placentae.

The adjusted logistic regression model showed an increased risk of severe adverse outcome for identified E-FGR pregnancies compared to non-identified SGA/FGR pregnancies, aOR 1.81, 95% CI 1.25–2.61. There was no association between identification of L-FGR pregnancies and severe adverse outcome, aOR 1.14, 95% CI 0.78–1.67.

**Fig 3:**
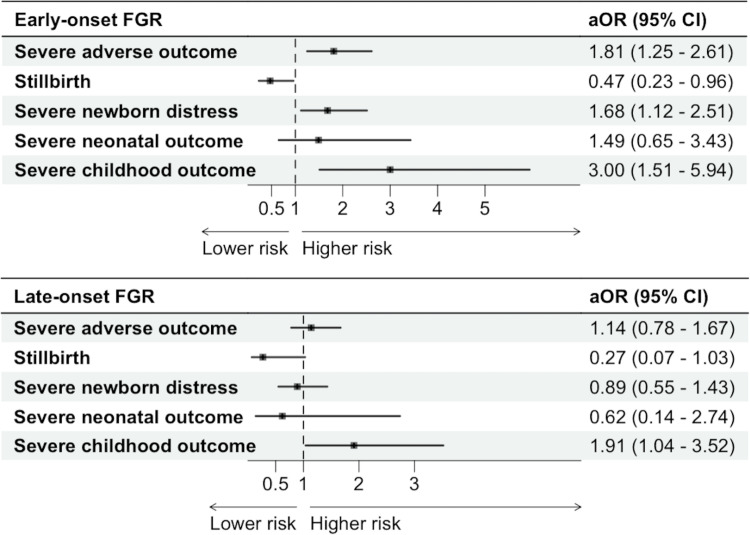
Adjusted odds ratios for the risk of severe adverse outcomes in identified early- and late onset FGR, compared to antenatally non-identified SGA/FGR. The odds ratios were adjusted for age, body mass index, smoking, education level, nulliparity, preeclampsia/hypertension, weight deviation at birth and preterm birth related diagnoses (at least one of the following; spontaneous preterm birth, preterm premature rupture of membranes, placenta previa, placenta accrete spectrum, ablatio placentae. FGR = fetal growth restriction, SGA = small for gestational age.

Analyzing subgroups of outcomes, we found a decreased risk of stillbirth for identified E-FGR compared to non-identified SGA/FGR, aOR 0.47, 95% CI 0.23–0.96, however, for L-FGR the association was not significant, aOR 0.27, 95% CI 0.07–1.03. The risk of severe newborn distress was increased for identified E-FGR fetuses compared to non-identified SGA/FGR, aOR 2.80, 95% CI 1.79–4.39. There was no significant association between antenatal identification of L-FGR and severe newborn distress, aOR 1.13, 95% CI 0.68–1.89. There was no significant association between identification of E-FGR or L-FGR and severe neonatal outcome, OR 1.49, 95% CI 0.65–3.43 and OR 0.62, 95% CI 0.14–2.74. The risk of severe childhood outcome was increased in identified E-FGR, OR 3.00, 95% CI 1.51–5.94 and in identified L-FGR, OR 1.91, 95% CI 1.04–3.52, compared to non-identified SGA/FGR. Adjusting for weight deviation at birth, preeclampsia/hypertension and preterm birth associated diagnoses affected the crude associations by far the most. The association between E-FGR and stillbirth even changed direction when adjusting for weight deviation at birth. Adding maternal age, BMI, smoking, educational level and parity into the regression model only had small effects on the results. Numbers and proportions of the included outcomes as well as crude and adjusted ORs for E-FGR and L-FGR, with non-identified SGA/FGR as the reference for E-FGR, and non-identified SGA/FGR with gestational age at delivery > 32 + 0 as the reference for L-FGR, are presented in Table 2 and illustrated in Fig 3.

**Table 2:** Severe adverse outcomes in identified early- and late-onset FGR, compared to antenatally non-identified SGA/FGR.

	Non- identified SGA/FGR	Identified early FGR					Non-identified SGA/FGR GA at birth > 32 + 0 w (ref)	Identified late FGR				
	(ref)		Crude		Adjusted				Crude		Adjusted	
	*n* = 3649	*n *= 308	OR	95% CI	OR^**^	95% CI	*n *= 3559	*n *= 722	OR	95% CI	OR^**^	95% CI
**Severe adverse outcome** ^*^	**220 (6.0)**	**73 (23.7)**	**4.84**	**3.60 - 6.51**	**1.81**	**1.25 - 2.61**	**151 (4.2)**	**49 (6.8)**	**1.64**	**1.18 - 2.29**	**1.14**	**0.78 - 1.67**
**Stillbirth**	**60 (1.6)**	**16 (5.2)**	**3.28**	**1.86 - 5.76**	**0.47**	**0.23 - 0.96**	**23 (0.6)**	**3 (0.4)**	**0.64**	**0.19 - 2.14**	**0.27**	**0.07 - 1.03**
**Severe newborn distress** ^*^	**105 (2.9)**	**46 (14.9)**	**5.93**	**4.10 - 8.57**	**2.80**	**1.79 - 4.39**	**78 (2.2)**	**27 (3.7)**	**1.73**	**1.11 - 2.71**	**1.13**	**0.68 - 1.89**
APGAR <4 at 5 minutes	8 (0.2)	4 (1.3)					4 (0.1)	2 (0.3)				
Umbilical pH < 7.0	24 (0.7)	2 (0.6)					24 (0.7)	4 (0.6)				
CPR > 10 minutes	82 (2.2)	45 (14.6)					55 (1.5)	23 (3.2)				
**Severe neonatal outcome** ^*^	**30 (0.8)**	**12 (3.9)**	**4.89**	**2.48 - 9.65**	**1.49**	**0.65 - 3.43**	**12 (0.3)**	**3 (0.4)**	**1.23**	**0.35 - 4.38**	**0.62**	**0.14 - 2.74**
HIE 2–3	2 (0.1)	1 (0.3)					2 (0.1)	1 (0.1)				
Necrotizing enterocolitis	2 (0.1)	6 (1.9)					0 (0.0)	1 (0.1)				
Neonatal seizures	7 (0.2)	1 (0.3)					4 (0.1)	1 (0.1)				
IVH 3–4	4 (0.1)	0 (0.0)					1 (0.1)	1 (0.1)				
BPD	12 (0.3)	7 (2.3)					0 (0.0)	0 (0.0)				
Infant death (< 1 y)	12 (0.3)	1 (0.3)					7 (0.2)	1 (0.1)				
**Severe childhood outcome** ^*^	**46 (1.3)**	**15 (4.9)**	**4.01**	**2.21 - 7.27**	**3.00**	**1.51 - 5.94**	**43 (1.2)**	**18 (2.5)**	**2.09**	**1.20 - 3.65**	**1.91**	**1.04 - 3.52**
Cognitive impairment	17 (0.5)	7 (2.3)					17 (0.5)	6 (0.8)				
Motor impairment	9 (0.2)	2 (0.6)					9 (0.3)	6 (0.8)				
Cerebral palsy	6 (0.2)	2 (0.6)					3 (0.1)	1 (0.1)				
Hearing impairment	17 (0.5)	3 (1.0)					17 (0.5)	9 (1.2)				
Visual impairment	0 (0.0)	2 (0.6)					0 (0.0)	0 (0.0)				
Childhood death (1-3y)	2 (0.1)	1 (0.3)					2 (0.1)	3 (0.4)				

SGA = small for gestational age, BPD = Bronchopulmonary dysplasia, HIE = Hypoxic ischemic encephalopathy, IVH = intraventricular hemorrhage, CPR = resuscitation activities, OR = odds ratio, CI = confidence interval, unclassified = unable to diagnose as SGA or FGR due to incomplete Doppler examinations.

* At least one of the below diagnoses.

** Adjusted for age, body mass index, smoking, education level, nulliparity, preeclampsia/hypertension, weight deviation at birth and preterm birth related diagnoses (at least one of the following: spontaneous preterm birth, preterm premature rupture of membranes, placenta previa, placenta accrete spectrum, ablatio placentae. Weight deviation at birth was categorized as ≤ −15% - > −22% (ref), −22,1% - > −28%, −28,1% - > −33%, > −33%. Education level was categorized as ≤ 9y, 10-12y, < 12y (ref). Body mass index was categorized as < 18.5, 18.5–24.9, 25.0–30.0, > 30.0. Age was categorized as > 35 years yes/no, parity as nulliparous yes/no, smoking as yes/no and preeclampsia/hypertension as yes/no

Numbers and proportions of the included outcomes and crude and adjusted ORs for the risk of adverse outcomes in fetuses classified as identified SGA and identified UC, compared to non-identified SGA/FGR, are presented in S3 Table.

No interactions were found between any of the variables included in the analyses.

There were missing data on BMI, smoking and educational level. Numbers and proportions of individuals with missing data on these variables, in exposed and non-exposed, are presented in Table 1. The proportion of cases that had missing data on one or more variables was 11.8%. Results of the sensitivity analyses for the handling of missing data are presented in the S4 Table. For sensitivity analyses of the regression models, 1) without categorizing the continuous variables and 2) only including covariates that significantly affected the associations, see S5 Table.

The interaction analysis, investigating the introduction of a mandatory evaluation of CPR in late SGA pregnancies, showed no significant modifying effect of year of birth on the association between identification of SGA delivered > 32 weeks of pregnancy, and severe adverse outcomes, *p* = 0.593 for severe adverse outcome, *p* = 0.126 for stillbirth, *p* = 0.854 for severe newborn distress, *p* = 0.902 for severe neonatal outcome and *p* = 0.544 for severe childhood outcome.

## Discussion

In this register-based cohort study of SGA pregnancies, identification of E-FGR was associated with a decreased risk of stillbirth, but an increased risk of severe newborn distress and severe childhood outcome, compared to non-identified SGA/FGR. Identified L-FGR had an increased risk for severe childhood outcome, compared to non-identified SGA/FGR, but no significant association was found between identification of L-FGR and stillbirth, severe newborn distress or severe neonatal outcome.

The absolute numbers of severe adverse outcomes in the overall SGA population were low, however identified E-FGR fetuses were clearly the most affected. These results are in agreement with previous research suggesting that E-FGR represents the more severe form of the disease, which often entails severely deprived fetuses despite careful surveillance [25]. The increased risk of the composite outcome in identified E-FGR compared to non-identified SGA/FGR pregnancies might be explained by the fact that severe SGA, which often turns out to be E-FGR, was identified to a higher degree. This is in agreement with Relph et al. who concluded that the probability of being identified as SGA/FGR is higher in severe SGA [27].

Previous studies comparing identified to non-identified SGA/FGR, do not separate E-FGR from L-FGR [6,7,28]. Gardosi et al. showed a 5-fold increased risk of stillbirth in non-identified SGA/FGR, compared to identified SGA [6]. In the current study, identified E-FGR, in contrast to L-FGR, had a decreased risk for stillbirth compared to non-identified SGA/FGR. The only just not significant result for the risk of stillbirth in identified L-FGR might be explained by a type-2 error; the mortality of L-FGR is low [25]. However, other explanations must be considered. L-FGR is challenging to predict and identify before birth; it is weakly associated with preeclampsia and the weight deviation is often modest [25,29,30]. A large proportion of L-FGR fetuses will probably remain non-identified. In addition, we still lack evidence to choose the right time point for delivery for L-FGR [31]. In late pregnancy, the fetal demand of the placenta is predominantly respiratory and abnormalities in the uterine artery Doppler are rare [32,33]. Without a reliable marker for impending death or deterioration, the decision to deliver might come too late or unnecessarily early. Our study shows that, for the surviving fetuses, identified E-FGR as well as L-FGR, had an increased risk for severe childhood outcome compared to non-identified SGA/FGR fetuses. This indicates that non-identified SGA/FGR who survive in utero are mild cases with mostly good outcomes, while non-identified *severe* SGA/FGR cases have a higher risk of dying in utero. Prematurity and low birth weight are independent risk factors for perinatal morbidity and mortality [34]. Our results were adjusted for weight-deviation at birth, but not for gestational age at delivery, suggesting that the increased risk for severe childhood outcome might be explained by induced preterm or early term delivery. Identified L-FGR fetuses, as well as E-FGR fetuses, had a shorter gestational age at delivery, compared to non-identified SGA/FGR fetuses. However, it is impossible to tell whether it is the immaturity or the harmful environment causing the prematurity that has the largest impact [35]. Hence, our results might as well be explained by the fact that we identified the “true” vulnerable fetuses, marked for life by a harmful intrauterine environment. In E-FGR, the risk benefit ratio, assessing the risk of stillbirth versus the chance of survival without major morbidity, per gestational week, is well established [9,36,37]. Therefore, a certain prevalence of severe childhood outcome is accepted and expected in this group. For L-FGR, the situation is different. The mortality of L-FGR is low [25], which is confirmed in our data. Immediate induction of late SGA pregnancies > 36 weeks of gestation did not decrease the risk of adverse outcomes, compared to expectant management [10]. In addition, previous studies have shown that children born early term or late preterm are at increased risk for cognitive disorders, compared to children born at 39 + 0–41 + 0 weeks of gestation [12,38].

In the present study, identified E-FGR fetuses had an increased risk for severe newborn distress compared to non-identified SGA/FGR, whereas no association was found between identified L-FGR and severe newborn distress. This finding probably reflects the high detection rate and the severity of the disease of E-FGR and the fact that E-FGR is often delivered preterm. Despite a timed and planned delivery, identified E-FGR fetuses, unlike L-FGR fetuses, will often be distressed directly after delivery. We found no significant associations between antenatal identification of E-FGR or L-FGR and severe neonatal outcome, which might be explained by the rarity of these events and the lack of power.

Universal screening with routine ultrasounds in weeks 28–30 and 34–36 increases the detection rate of SGA but does not improve perinatal outcome, compared with selective screening, i.e., ultrasound is performed only if clinically indicated [39]. Hence, universal screening with ultrasound does not perform better than selective screening in finding vulnerable SGA fetuses in low-risk pregnancies. This does not contradict the fact that non-identified SGA/FGR are at increased risk of demise and should be paid attention to [8]. The failure in the effectiveness of ultrasound screening has been addressed [27,32]. At the beginning of the 21th century, CPR was introduced as an additional examination for the evaluation of late SGA pregnancies. A low CPR is associated with poor outcomes, although its predictive value has been questioned [31,40–45]. In the present study, the introduction of a mandatory evaluation of the CPR in late SGA pregnancies did not influence the associations between identification of SGA and severe adverse outcomes.

The primary strength of this study is the near population-based cohort, enabling a high external validity, provided a similar setting. We used nationwide registers with high coverage and validity and had access to all desirable variables which allowed for a careful confounder adjustment. The data was collected prospectively. In addition, a meticulous evaluation of all ultrasound reports was performed in order to categorize fetuses correctly, in accordance with the Delphi agreement on the definition of FGR [24]. Gestational age at delivery was not considered a confounder but a mediator since ending the pregnancy is a crucial part of the management of FGR [35]. Outcome data were chosen according to an international consensus document and analyzed as a broad composite outcome as well as in prespecified subgroups of timely linked outcomes [26].

The major limitation of the study is the unavoidable heterogenicity of the comparison group of non-identified SGA/FGR. The comparison group consists of a mixture of FGR and SGA fetuses. In addition, a selective screening was used to identify fetuses at risk of FGR. Consequently, the comparison group of non-identified SGA/FGR fetuses mainly consisted of less severely affected SGA fetuses [27]. Despite a solid adjustment strategy, residual confounding may exist. For L-FGR, the comparison group of non-identified SGA/FGR was restricted to include only fetuses delivered after gestational week 32 + 0 since fetuses delivered before that under no circumstances could be defined as L-FGR [24]. The comparison group for E-FGR was not restricted because E-FGR fetuses may be delivered in late pregnancy. However, there will still be some overlapping of E-FGR and L-FGR in the control group. We strictly followed the consensus criteria for the definition of E-FGR and L-FGR. Identified SGA fetuses with incomplete Doppler examinations, where a. cerebri media was the most frequently missing, were classified as “unclassified” and analyzed separately. The proportion of unclassified cases was not negligible, introducing a risk of misclassification bias which might strengthen the associations, especially for L-FGR. Severe adverse outcomes are rare, and the absolute number of the outcomes included in the subgroups is small. The manual review of the ultrasound reports limited the size of the study population. Furthermore, we had to exclude subjects with missing data on birthweight, gestational age at birth and those with no or invalid PIN, which could introduce bias. Available data of these subjects were evaluated, and the risk of informative bias was estimated as low [8].

## Conclusions

In this register-based study of 5499 SGA newborns, E-FGR fetuses benefited from identification with a decreased risk of stillbirth, however at the expense of an increased risk of severe newborn and childhood outcomes. Identification of L-FGR was associated with an increased risk for severe childhood outcome, while the negative association with stillbirth did not reach significance. The introduction of CPR in the management of late SGA pregnancies did not influence the associations between identification and severe adverse outcomes.

## Supporting information

S1 Appendix Screening and management of small for gestational age and fetal growth restriction: An English summary of Stockholm-based guidelines. (DOCX)

S1 Fig Directed acyclic graph of the assumptions about the relationship between variables and calculated minimal sufficient adjustment set. (DOCX)

S1 Table Consensus-based definitions for early- and late-onset fetal growth restriction, in absence of congenital anomalies. (DOCX)

S2 Table Background characteristics and major outcome groups for antenatally non-identified SGA compared to fetuses identified with ultrasound as “true” SGA or “unclassified”. (DOCX)

S3 Table Presence and risk of severe adverse outcomes in identified SGA and identified unclassified SGA, compared to antenatally non-identified SGA. (DOCX)

S4 Table Sensitivity analysis on the handling of missing data. The results of the chosen model, i.e., missing data as a separate category (aOR), and models with imputed best case (aORbc) and worst case (aORwc) values, and a model with a complete case analysis (aORcc).
(DOCX)

S5 Table Sensitivity analyses of the logistic regression model. Results of 1) a model only including the most influential confounding variables 2) a model with continuous variables not categorized 3) the chosen model, which included all identified confounders, with continuous variables categorized.
(DOCX)
