# Supplementary material for: Antenatal identification of early- and late-onset fetal growth restriction and the possible impact of the introduction of cerebroplacental ratio: Effect on perinatal and childhood outcome
Source: PLoS One. 2025 Jun 18;20(6):e0325906. doi: 10.1371/journal.pone.0325906 (PMC12176146; doi:10.1371/journal.pone.0325906)
Supplement: S1 Appendix — (DOCX) [file pone.0325906.s001.docx]

**S1 Appendix. Screening and management of small for gestational age and fetal growth restriction: An English summary of Stockholm-based guidelines.**

The Stockholm Region uses a risk screening regime to identify fetal growth restriction (FGR). Only high-risk pregnancies are examined with ultrasound repeatedly during pregnancy. Repeated measurements of the symphyses-fundal height are the main factor used for the screening/identification of FGR. If a deviation in the symphysis-fundal height curve is found or if pregnancy complications occur, an ultrasound is performed. Estimated fetal weight, which is based on bi-parietal diameter, abdominal diameter and femur length, is calculated, and a weight deviation is obtained based on a longitudinal population-based growth chart.^1, 2^ An estimated fetal weight of > −15% is considered normal.

If an ultrasound examination results in an estimated fetal weight of ≤ −15%, which corresponds to the < 10^th^ percentile, Doppler examinations of the umbilical artery and the uterine arteries are performed. A pulsatility index (PI) in the umbilical artery of > mean + 2 standard deviations (SD), is considered abnormal.^3^ The uterine artery is examined bilaterally, and the mean PI is calculated. Reference ranges published by Gomez et al. are used; values greater than the 95^th^ centile are considered abnormal.^4^ If the fetus is 34 weeks or older, a Doppler examination of a. cerebri media should be performed, and a cerebroplacental ratio (CPR) is calculated^*^.^5^ A CPR less than the 5^th^ percentile is considered abnormal. If an abnormality is found in the Doppler examination or a pronounced weight deviation is observed, further Doppler examinations and a careful evaluation of the anatomy of the fetus are required; otherwise, a follow-up ultrasound should be performed in 2 weeks.

The following applies for when to deliver patients with FGR: In early pregnancies (< 32 weeks) an individual approach, which is based on the TRUFFLE recommendations, is recommended.^6^ If the pregnancy is more than 32 weeks and the Doppler examination shows absent or reversed end-diastolic blood flow in the umbilical artery, delivery should be considered. In more than 34 weeks of pregnancy, delivery is considered if the PI in the umbilical artery is > mean + 3 SD, using the Swedish score system for blood flow resistance in the umbilical artery.^7^ In more than 37 + 0 weeks of pregnancy, a PI in the umbilical artery of > mean + 2 SD implies recommendations to start the delivery. In pregnancies with normal Doppler examination in the umbilical artery but with redistribution (abnormal CPR), expectation is recommended until 38 weeks, and up to 40 weeks provided that all other parameters (fetal movements, amniotic fluid and maternal health) are normal and weekly check-ups are performed. Cardiotocography (CTG) is performed regularly on all suspected FGR fetuses, and if pathologic, delivery is recommended regardless of the Doppler values.

The guidelines do not cover recommendations on mode of delivery.

* Mandatory from late 2015

1 Marsal K, Persson PH, Larsen T, Lilja H, Selbing A, Sultan B. Intrauterine growth curves based on ultrasonically estimated foetal weights. Acta Paediatr. 1996; 85(7): 843-8.

2 Persson PH, Weldner BM. Intra-uterine weight curves obtained by ultrasound. Acta Obstet Gynecol Scand. 1986; 65(2): 169-73.

3 Gudmundsson S, Marsál K. Umbilical artery and uteroplacental blood flow velocity waveforms in normal pregnancy--a cross-sectional study. Acta Obstet Gynecol Scand. 1988; 67(4): 347-54.

4 Gómez O, Figueras F, Fernández S, Bennasar M, Martínez JM, Puerto B, et al. Reference ranges for uterine artery mean pulsatility index at 11-41 weeks of gestation. Ultrasound Obstet Gynecol. 2008; 32(2): 128-32.

5 Ebbing C, Rasmussen S, Kiserud T. Middle cerebral artery blood flow velocities and pulsatility index and the cerebroplacental pulsatility ratio: Longitudinal reference ranges and terms for serial measurements. Ultrasound Obstet Gynecol. 2007; 30(3): 287-96.

6 Lees CC, Marlow N, van Wassenaer-Leemhuis A, Arabin B, Bilardo CM, Brezinka C, et al. 2 year neurodevelopmental and intermediate perinatal outcomes in infants with very preterm fetal growth restriction (TRUFFLE): A randomised trial. Lancet. 2015; 385(9983): 2162-72.

7 Gudmundsson S, Korszun P, Olofsson P, Dubiel M. New score indicating placental vascular resistance. Acta Obstet Gynecol Scand. 2003; 82(9): 807-12.
