# Supplementary material for: Antenatal identification of early- and late-onset fetal growth restriction and the possible impact of the introduction of cerebroplacental ratio: Effect on perinatal and childhood outcome
Source: PLoS One. 2025 Jun 18;20(6):e0325906. doi: 10.1371/journal.pone.0325906 (PMC12176146; doi:10.1371/journal.pone.0325906)
Supplement: S1 Fig — (DOCX) [file pone.0325906.s002.docx]

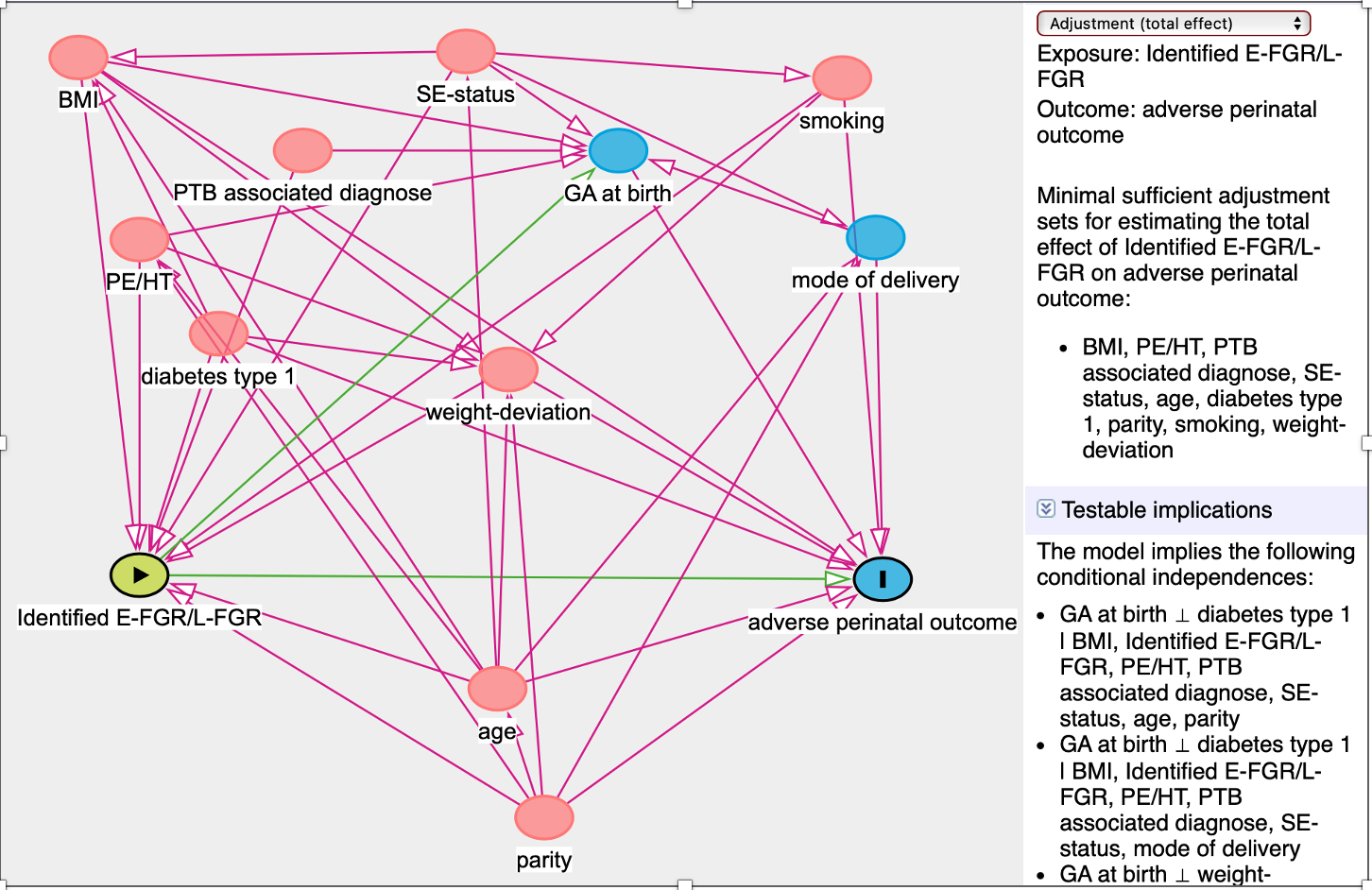


**S1 Fig.** Directed acyclic graph of the assumptions about the relationship between variables and calculated minimal sufficient adjustment set (dagitty.net). SGA = small for gestational age, PE = preeclampsia, HT = hypertension, BMI = body mass index, SE = socioeconomic, GA = gestational age. Weight deviation = deviation from the expected (mean) weight by gestational age.
