# Supplementary material for: Antenatal identification of early- and late-onset fetal growth restriction and the possible impact of the introduction of cerebroplacental ratio: Effect on perinatal and childhood outcome
Source: PLoS One. 2025 Jun 18;20(6):e0325906. doi: 10.1371/journal.pone.0325906 (PMC12176146; doi:10.1371/journal.pone.0325906)
Supplement: S1 Table — (DOCX) [file pone.0325906.s003.docx]

**S1 Table.** Consensus-based definitions for early- and late-onset fetal growth restriction (FGR) in absence of congenital anomalies (25)

| Early FGR:  GA < 32 weeks, in absence of congenital anomalies | Late FGR:  GA ≥ 32 weeks, in absence of congenital anomalies |
| --- | --- |
| AC/EFW < 3^rd^ centile or UA-AEDF  Or   1. AC/EFW < 10^th^ centile combined with 2. UtA-PI > 95^th^ centile and/or 3. UA-PI > 95^th^ centile | AC/EFW < 3^rd^ centile  Or at least two out of three of the following   1. AC/EFW < 10^th^ centile 2. AC/EFW crossing centiles > 2 quartiles on growth centiles* 3. CPR < 5^th^ centile or UA-PI > 95^th^ centile |

*Growth centiles are non-customized centiles, AC, fetal abdominal circumference; AEDF, absent end-diastolic flow; CPR, cerebroplacental ratio; EFW, estimated fetal weight; GA, gestational age; PI, pulsatility index; UA, umbilical artery, UtA, uterine artery.
