# Supplementary material for: Antenatal identification of early- and late-onset fetal growth restriction and the possible impact of the introduction of cerebroplacental ratio: Effect on perinatal and childhood outcome
Source: PLoS One. 2025 Jun 18;20(6):e0325906. doi: 10.1371/journal.pone.0325906 (PMC12176146; doi:10.1371/journal.pone.0325906)
Supplement: S2 Table — (DOCX) [file pone.0325906.s004.docx]

| **S2 Table. Background characteristics and major outcome groups for non-identified SGA/FGR compared to identified SGA and identified unclassified fetuses.** | | | | | | | |
| --- | --- | --- | --- | --- | --- | --- | --- |
|  |  |  | **Non-ID SGA/FGR** (reference) | **ID SGA** |  | **ID unclassified** |  |
|  |  |  | n=3649 | n = 225 | p-value | n = 595 | p-value |
| **Maternal characteristics** | | |  |  |  |  |  |
|  | Age (years) | | 32.0 (28.0, 35.0) | 31.0 (28.0, 35.0) | 0.999 | 32.0 (28.0, 35.0) | 0.797 |
|  |  | >35 | 1062 (29.1) | 63 (28.0) | 0.723 | 169 (28.4) | 0.727 |
|  | BMI (kg/m^2^) | | 22.8 (20.8, 25.7) | 22.7 (20.4, 25,4) | 0.155 | 22.1 (20.4, 25.0) | <.001 |
|  |  | <18.5 | 127 (3.5) | 12 (5.3) | 0.193 | 37 (6.2) | 0.005 |
|  |  | 18.5-24.9 | 2268 (62.2) | 136 (60.4) |  | 382 (64.2) |  |
|  |  | 25-30 | 751 (20.6) | 47 (20.9) |  | 98 (16.5) |  |
|  |  | >30 | 279 (7.6) | 11 (4.9) |  | 45 (7.6) |  |
|  |  | missing | 224 (6.1) | 19 (8.4) |  | 33 (5.5) |  |
|  | Smoking (yes) | | 163 (4.5) | 10 (4.4) | 0.813 | 33 (5.5) | 0.485 |
|  |  | missing | 191 (5.2) | 14 (6.2) |  | 29 (4.9) |  |
|  | Nullipara (yes) | | 2249 (61.6) | 124 (55.1) | 0.051 | 340 (57.1) | 0.037 |
|  | Diabetes (yes) | | 16 (0.4) | 5 (2.2) | 0.006 | 5 (0.8) | 0.203 |
|  | Education | |  |  |  |  |  |
|  |  | < 9 years | 385 (10.6) | 18 (8.0) | 0.344 | 62 (10.4) | 0.860 |
|  |  | 10 to 12 years | 1028 (28.2) | 56 (24.9) |  | 169 (28.4) |  |
|  |  | >12 years | 2108 (57.8) | 142 (63.1) |  | 339 (57.0) |  |
|  |  | missing | 128 (3.5) | 9 (4.0) |  | 25 (4.2) |  |
| **Start of delivery** | | |  |  |  |  |  |
|  | Spontaneous | | 2600 (71.3) | 139 (61.8) | 0.002 | 371 (62.4) | <.001 |
|  | Induction | | 784 (21.5) | 65 (28.9) |  | 160 (26.9) |  |
| **Mode of delivery** | | |  |  |  |  |  |
|  | Spontaneous vaginal | | 2698 (73.9) | 160 (71.1) | 0.207 | 430 (72.3) | 0.027 |
|  | Instrumental vaginal | | 325 (8.9) | 15 (6.7) |  | 47 (7.9) |  |
|  | Elective cesarean | | 154 (4.2) | 17 (7.6) |  | 52 (8.7) |  |
|  | Emergency cesarean | | 472 (12.9) | 33 (14.7) |  | 66 (11.1) |  |
| **Pregnancy complication** | | |  |  |  |  |  |
|  | Hypertension | | 133 (3.6) | 16 (7.1) | 0.009 | 28 (4.7) | 0.209 |
|  | PE/HELLP | | 190 (5.2) | 16 (7.1) | 0.217 | 31 (5.2) | 0.997 |
|  | PTB associated diagnose** | | 171 (4.7) | 18 (8.0) | 0.025 | 29 (4.9) | 0.841 |
| **Pregnancy outcome** | | |  |  |  |  |  |
|  | GA at delivery (days) | | 281 (274, 287) | 280 (271, 286) | 0.019 | 281 (274, 288) | 0.121 |
|  | Birthweight (gram) | | 2900 (2702, 3065) | 2800 (2583, 2958) | <.001 | 2880 (2690, 3030) | 0.181 |
|  | Birthweight deviation (%) | | -18.4 (-21.6, -16.5) | -20.3 (-23.5, -18.0) | <.001 | -19.3 (-22.6, -17.1) | <.001 |
| **Severe adverse outcome*** | | |  |  |  |  |  |
|  | Severe adverse outcome** | | 220 (6.0) | 8 (3.6) | 0.126 | 18 (3.0) | 0.003 |
|  |  | Stillbirth | 60 (1.6) | 0 (0.0) | 0.048 | 1 (0.2) | 0.005 |
|  |  | Severe newborn distress | 105 (2.9) | 6 (2.7) | 0.854 | 10 (1.7) | 0.095 |
|  |  | Severe neonatal outcome | 30 (0.8) | 4 (1.8) | 0.132 | 0 (0.0) | 0.016 |
|  |  | Severe childhood outcome | 46 (1.3) | 1 (0.4) | 0.522 | 8 (1.3) | 0.866 |

Continuous variables are presented as medians with interquartile range, categorical variables as numbers and proportions. SGA = small for gestational age,

FGR = fetal growth restriction, Non-ID SGA = non-identified as ≤ -15% before birth, ID = identified as, unclassified = unable to diagnose as SGA or FGR due to incomplete Doppler examinations, according to Gordijnj et al, BMI = body mass index, PE = preeclampsia, HELLP = Hemolytic Elevated Liver enzyme Low Platelet syndrome, GA = gestational age, PTB = preterm birth. * Major outcome groups ** At least one of the following; spontaneous preterm birth, preterm premature rupture of membranes, placenta previa, placenta accrete spectrum, ablatio placentae.
