# Supplementary material for: Antenatal identification of early- and late-onset fetal growth restriction and the possible impact of the introduction of cerebroplacental ratio: Effect on perinatal and childhood outcome
Source: PLoS One. 2025 Jun 18;20(6):e0325906. doi: 10.1371/journal.pone.0325906 (PMC12176146; doi:10.1371/journal.pone.0325906)
Supplement: S3 Table — (DOCX) [file pone.0325906.s005.docx]

| **S3 Table. Prevalence and risk of severe adverse outcomes in identified SGA and identified unclassified SGA, compared to antenatally** | | | | | | | | | | |
| --- | --- | --- | --- | --- | --- | --- | --- | --- | --- | --- |
| **non-identified SGA/FGR** | | |  |  |  |  |  |  |  |  |
|  |  |  | **Non-identified** | **Identified** |  |  | I**dentified** |  |  |  |
|  |  |  | **SGA/FGR** = ref | **SGA** | Crude | Adjusted** | **unclassified** | Crude | Adjusted** |  |
|  |  |  | n = 3649 | n = 225 | OR (95% CI) | OR (95% CI) | n = 595 | OR (95% CI) | OR (95% CI) |  |
|  |  |  |  |  |  |  |  |  |  |  |
| **Severe adverse outcome*** | | | **220 (6.0)** | **8 (3.6)** | **0.58 (0.28 - 1.18)** | **0.51 (0.25 - 1.08)** | **18 (3.0)** | **0.49 (0.30 - 0.79)** | **0.51 (0.31 - 0.83)** |  |
|  |  |  |  |  |  |  |  |  |  |  |
|  | **Stillbirth** | | **60 (1.6)** | **0 (0.0)** | **0.00** | **0.00** | **1 (0.2)** | **0.10 (0.01 - 0.73)** | **0.13 (0.02 - 0.95)** |  |
|  |  |  |  |  |  |  |  |  |  |  |
|  | **Severe newborn distress*** | | **105 (2.9)** | **6 (2.7)** | **0.93 (0.40 - 2.13)** | **0.75 (0.32 - 1.77)** | **10 (1.7)** | **0.58 (0.30 – 1.11)** | **0.58 (0.30 – 1.14)** |  |
|  |  | APGAR <4 at 5 minutes | 8 (0.2) | 1 (0.4) |  |  | 2 (0.3) |  |  |  |
|  |  | Umbilical pH <7.0 | 24 (0.7) | 0 (0.0) |  |  | 3 (0.5) |  |  |  |
|  |  | CPR > 10 minutes | 82 (2.2) | 6 (2.7) |  |  | 6 (1.0) |  |  |  |
|  |  |  |  |  |  |  |  |  |  |  |
|  | **Severe neonatal outcome*** | | **30 (0.8)** | **4 (1.8)** | **2.18 (0.76 - 6.25)** | **1.86 (0.61 - 5.65)** | **0 (0.0)** | **0.00** | **0.00** |  |
|  |  | HIE 2 to 3 | 2 (0.1) | 0 (0.0) |  |  | 0 (0.0) |  |  |  |
|  |  | Necrotizing enterocolitis | 2 (0.1) | 1 (0.4) |  |  | 0 (0.0) |  |  |  |
|  |  | Neonatal seizures | 7 (0.2) | 0 (0.0) |  |  | 0 (0.0) |  |  |  |
|  |  | IVH 3 to 4 | 4 (0.1) | 1 (0.4) |  |  | 0 (0.0) |  |  |  |
|  |  | BPD | 12 (0.3) | 2 (0.9) |  |  | 0 (0.0) |  |  |  |
|  |  | Infant death (< 1 y) | 12 (0.3) | 1 (0.4) |  |  | 0 (0.0) |  |  |  |
|  |  |  |  |  |  |  |  |  |  |  |
|  | **Severe childhood outcome*** | | **46 (1.3)** | **1 (0.4)** | **0.35 (0.05 - 2.55)** | **0.34 (0.05 - 2.46)** | **8 (1.3)** | **1.07 (0.50 - 2.27)** | **1.06 (0.50 - 2.27)** |  |
|  |  | Cognitive impairment | 17 (0.5) | 1 (0.4) |  |  | 8 (1.3) |  |  |  |
|  |  | Motor impairment | 9 (0.2) | 0 (0.0) |  |  | 1 (0.2) |  |  |  |
|  |  | Cerebral palsy | 6 (0.2) | 0 (0.0) |  |  | 0 (0.0) |  |  |  |
|  |  | Hearing impairment | 17 (0.5) | 0 (0.0) |  |  | 0 (0.0) |  |  |  |
|  |  | Visual impairment | 0 (0.0) | 0 (0.0) |  |  | 0 (0.0) |  |  |  |
|  |  | Childhood death (1-3y) | 2 (0.1) | 0 (0.0) |  |  | 0 (0.0) |  |  |  |
|  |  |  |  |  |  |  |  |  |  |  |

SGA = small for gestational age, FGR = fetal growth restriction, BPD = Bronchopulmonary dysplasia, HIE = Hypoxic ischemic encephalopathy, IVH = intraventricular hemorrhage, CPR = resuscitation activities, OR = odds ratio, CI = confidence interval, unclassified = unable to diagnose as SGA or FGR due to incomplete doppler examinations. *At least one of the below diagnoses **Adjusted for age, body mass index, smoking, education level, nulliparity, preeclampsia/hypertension, weight deviation at birth and preterm birth (PTB) associated diagnose, i.e., at least one of the following; spontaneous preterm birth, preterm premature rupture of membranes, placenta previa, placenta accrete spectrum, ablatio placentae. Weight deviation at birth was categorized as ≤ -15% -> -22% (ref), -22,1% -> -28%, -28,1% -> -33%, > -33%, education level was categorized as ≤ 9y, 10-12y, <12y (ref), body mass index was categorized as < 18.5, 18.5-24.9, 25.0-30.0, >30.0, age was categorized as > 35 years yes/no, parity as nulliparous yes/no, smoking as yes/no, preeclampsia/hypertension as yes/no and PTB associated diagnose as yes/no.
