# Supplementary material for: Antenatal identification of early- and late-onset fetal growth restriction and the possible impact of the introduction of cerebroplacental ratio: Effect on perinatal and childhood outcome
Source: PLoS One. 2025 Jun 18;20(6):e0325906. doi: 10.1371/journal.pone.0325906 (PMC12176146; doi:10.1371/journal.pone.0325906)
Supplement: S4 Table — The results of the chosen model, i.e., missing data as a separate category (aOR), and models with imputed best case (aORbc) and worst case (aORwc) values, and a model with a complete case analysis (aORcc). (DOCX) [file pone.0325906.s006.docx]

| **S4 Table. Sensitivity analysis on the handling of missing data; results of the chosen model, i.e., missing data as a separate categories (aOR), models with imputed best case (aORbc) and worst case (aORwc) values, and a model with a complete case analysis (aORcc).** | | | | | | | | |
| --- | --- | --- | --- | --- | --- | --- | --- | --- |
|  | | | | | | | | |
|  | **aOR** | **95% CI** | **aORbc** | **95% CI** | **aORwc** | **95% CI** | **aORcc** | **95% CI** |
| **Severe adverse outcome** |  |  |  |  |  |  |  |  |
| ID early | 1.81 | 1.25 - 2.61 | 1.84 | 1.28 - 2.66 | 1.80 | 1.25 - 2.59 | 1.95 | 1.30 - 2.92 |
| ID late | 1.14 | 0.78 - 1.67 | 1.15 | 0.79 - 1.68 | 1.14 | 0.78 - 1.67 | 1.14 | 0.75 - 1.73 |
| **Stillbirth** |  |  |  |  |  |  |  |  |
| ID early | 0.47 | 0.23 - 0.96 | 0.48 | 0.24 - 0.96 | 0.47 | 0.23 - 0.94 | 0.42 | 0.18 - 0.97 |
| ID late | 0.27 | 0.07 - 1.03 | 0.25 | 0.07 - 0.95 | 0.25 | 0.07 - 0.96 | 0.36 | 0.09 - 1.42 |
| **Severe newborn distress** |  |  |  |  |  |  |  |  |
| ID early | 2.80 | 1.79 - 4.39 | 2.81 | 1.80 - 4.40 | 2.74 | 1.75 - 4.29 | 3.03 | 1.85 - 4.94 |
| ID late | 1.13 | 0.68 - 1.89 | 1.14 | 0.69 - 1.90 | 1.14 | 0.68 - 1.89 | 0.96 | 0.55 - 1.69 |
| **Severe neonatal outcome** |  |  |  |  |  |  |  |  |
| ID early | 1.49 | 0.65 - 3.43 | 1.56 | 0.68 - 3.60 | 1.49 | 0.65 - 3.41 | 2.32 | 0.90 - 5.94 |
| ID late | 0.62 | 0.14 - 2.74 | 0.59 | 0.14 - 2.60 | 0.65 | 0.15 - 2.79 | 0.62 | 0.14 - 2.85 |
| **Severe childhood outcome** |  |  |  |  |  |  |  |  |
| ID early | 3.00 | 1.51 - 5.94 | 3.06 | 1.54 -6.06 | 3.04 | 1.53 - 6.01 | 3.06 | 1.41 - 6.66 |
| ID late | 1.91 | 1.04 - 3.52 | 1.91 | 1.04 - 3.53 | 1.91 | 1.03 - 3.51 | 2.19 | 1.12 - 4.25 |

aOR = adjusted odds ratio, missing values as separate categories, aORbc = adjusted odds ratio, missing values on body mass index (bmi) are categorized as bmi 18,5-24.9, missing values on smoking as non-smoker, and missing value on education level as > 12 years of education, aORwc = adjusted odds ratio, missing values on bmi are categorized as bmi > 30, missing values on smoking as smoker, and missing value on education level as < 9 years of education, aORcc = adjusted odds ratio, cases with missing values (11.8%) not included in the analyses (complete case analysis)
