# Supplementary material for: Antenatal identification of early- and late-onset fetal growth restriction and the possible impact of the introduction of cerebroplacental ratio: Effect on perinatal and childhood outcome
Source: PLoS One. 2025 Jun 18;20(6):e0325906. doi: 10.1371/journal.pone.0325906 (PMC12176146; doi:10.1371/journal.pone.0325906)
Supplement: S5 Table — Results of 1) a model only including the most influential confounding variables 2) a model with continuous variables not categorized 3) the chosen model, which included all identified confounders, with continuous variables categorized. (DOCX) [file pone.0325906.s007.docx]

| **S5 Table**. **Logistic regression models for the risk of severe adverse outcome, and subgroups of outcomes, in identified early - and late onset FGR compared to non-identified SGA/FGR** | | | | | | | | | | |  |
| --- | --- | --- | --- | --- | --- | --- | --- | --- | --- | --- | --- |
|  |  | Non-id SGA/FGR (ref) | **Early-onset FGR** | |  |  |  |  |  |  |  |
|  |  | % | % | cOR | CI | aOR1 | CI | aOR2 | CI | aOR3 | CI |
| Severe adverse outcome* | | 6.0 | 23.7 | 4.84 | 3.60 - 6.51 | 1.81 | 1.25 - 2.61 | 1.91 | 1.33 - 2.75 | 1.70 | 1.16 - 2.51 |
|  | Stillbirth | 1.6 | 5.2 | 3.28 | 1.86 - 5.76 | 0.47 | 0.23 - 0.96 | 0.49 | 0.25 - 0.97 | 0.28 | 0.10 - 0.73 |
|  | Severe newborn distress | 2.9 | 14.9 | 5.93 | 4.10 - 8.57 | 2.80 | 1.79 - 4.39 | 2.81 | 1.80 - 4.37 | 3.18 | 1.99 - 5.07 |
|  | Severe neonatal outcome | 0.8 | 3.9 | 4.89 | 2.48 - 9.65 | 1.49 | 0.65 - 3.43 | 1.90 | 0.83 - 4.36 | 2.80 | 1.16 - 6.76 |
|  | Severe childhood outcome | 1.3 | 4.9 | 4.01 | 2.21 - 7.27 | 3.00 | 1.51 - 5.94 | 3.28 | 1.67 - 6.43 | 2.79 | 1.33 - 5.87 |
|  |  |  |  |  |  |  |  |  |  |  |  |
|  |  | Non id SGA/FGR, GA at | **Late onset FGR** | |  |  |  |  |  |  |  |
|  |  | birth >32+0 weeks (ref) |  |  |  |  |  |  |  |  |  |
|  |  | % | % | cOR | CI | aOR1 | CI | aOR2 | CI | aOR3 | CI |
| Severe adverse outcome* | | 4.2 | 6.8 | 1.64 | 1.18 - 2.29 | 1.14 | 0.78 - 1.67 | 1.12 | 0.77 - 1.64 | 1.05 | 0.71 - 1.56 |
|  | Stillbirth | 0.6 | 0.4 | 0.64 | 0.19 - 2.14 | 0.27 | 0.07 - 1.03 | 0.23 | 0.06 - 0.88 | 0.30 | 0.08 - 1.11 |
|  | Severe newborn distress | 2.2 | 3.7 | 1.73 | 1.11 - 2.71 | 1.13 | 0.68 - 1.89 | 1.11 | 0.67 - 1.84 | 0.90 | 0.52 - 1.55 |
|  | Severe neonatal outcome | 0.3 | 0.4 | 1.23 | 0.35 - 4.38 | 0.62 | 0.14 - 2.74 | 0.59 | 0.14 - 2.54 | 0.77 | 0.19 - 3.22 |
|  | Severe childhood outcome | 1.2 | 2.5 | 2.09 | 1.20 - 3.65 | 1.91 | 1.04 - 3.52 | 1.90 | 1.03 - 3.49 | 1.99 | 1.07 - 3.70 |
|  |  |  |  |  |  |  |  |  |  |  |  |

* One or more of the below, OR = odds ratio, cOR = crude odds ratio, CI = confidence interval, FGR = fetal growth restriction, SGA = small for gestational age, aOR1 = original model, adjusted for all identified confounders, i.e., age, body mass index, smoking, education level, nulliparity, preeclampsia/hypertension, weight deviation at birth and PTB-associated diagnose (at least one of the following; spontaneous preterm birth, preterm premature rupture of membranes, placenta previa, placenta accrete spectrum, ablatio placentae), aOR2 = adjusted for preeclampsia/hypertension, PTB associated diagnose and weight deviation at birth, the confounders who had the largest effect on the associations, aOR 3 = adjusted for all, continuous variables not categorized.
